# Adaptive federated learning with differential privacy for multi-class respiratory disease recognition from lung sound recordings

**DOI:** 10.3389/fdgth.2026.1902226

**Published:** 2026-08-24

**Authors:** Syed Riyazul Haq, Kuruva Lakshmanna

**Affiliations:** School of Computer Science Engineering and Information Systems, Vellore Institute of Technology, Vellore, India

**Keywords:** acoustic feature extraction, adaptive federated aggregation, differential privacy, federated learning, non-IID partitioning, respiratory sound classification, SMOTE

## Abstract

**Introduction:**

The classification of respiratory sounds remains a challenging task in clinical research, limited by severe dataset imbalance and strict privacy regulations. Traditional machine learning methods are centralized and jeopardize data privacy and have difficulty in identifying minority classes, which makes them unsuitable for real-world clinical use.

**Methods:**

In this paper, a novel federated learning framework for multi-class respiratory disease classification is proposed based on the fusion of a Hybrid Support Vector Machine-K-Nearest Neighbor (SVMKNN) classifier with Adaptive Federated Aggregation (AFA), Synthetic Minority Oversampling Technique (SMOTE) balancing, and a Gaussian differential privacy (DP) mechanism. The suggested framework runs using five non-independently and identically distributed (non-IID) client nodes partitioned using Dirichlet distribution (α = 1.0) and able to perform privacy-preserving collaborative training without sharing raw data. We extract a rich multi-modal acoustic feature representation from respiratory recordings using Mel-Frequency Cepstral Coefficients (MFCCs), first and second order delta-MFCCs, Mel spectrograms, chroma features, spectral contrast, and a set of seven scalar acoustic descriptors. The resulting high-dimensional feature vector is compressed using Principal Component Analysis (PCA) to retain 95% cumulative variance. The hybrid classifier combines the discriminative boundary learning of SVM with the local density estimation of KNN through a weighted probability ensemble (α = 0.6), resulting in better generalization in federated, class-imbalanced settings. A major methodological innovation is the restriction of the differential privacy noise to the aggregation phase, so that all reported metrics are based on clean, noise-free model outputs.

**Result:**

The suggested framework obtains an accuracy of 97.55, a weighted F1-score of 97.64, Matthews Correlation Coefficient (MCC) of 90.89, and Cohen's Kappa of 90.77.

**Discussion:**

Ablation studies confirm that federated hybrid learning with AFA and SMOTE outperforms standalone SVM, standalone KNN, and centralized hybrid baselines for all evaluation metrics, proving the clinical viability of the proposed framework for distributed hospital networks and telemedicine platforms.

## 1 Introduction

Respiratory diseases are one of the most common public health problems worldwide, affecting hundreds of millions of people with chronic illness and still ranking among the leading causes of death. Respiratory morbidity such as Chronic Obstructive Pulmonary Disease (COPD), asthma, bronchiectasis, bronchiolitis, pneumonia, and upper respiratory tract infections place a large burden on health systems, especially in low-resource settings where specialist pulmonologists are not feasible together with diagnostic tools [1]. Timely treatment can influence the progression of a disease and the recovery of a patient; early and accurate diagnosis of respiratory diseases has been playing an important role. Clinically, auscultation (listening with a stethoscope to the lung sounds) has been used as the standard method of screening for respiratory disease for many [2]. However, it is not very reliable as it is dependent on the clinician, as skill and experience vary significantly among practitioners, and results can differ obviously from one clinician to the next and the method can be less readily available where specialists are less prevalent [3]. This has dramatically shifted with the introduction of wearable acoustic sensors and digital stethoscopes [4], which enable respiratory sounds to be analysed using machine learning and which allow for an objective clinical skill to be made more consistent, repeatable, and scalable. There are a number of studies that demonstrate this change.

The authors [5] proposed that sequence-to-sequence recurrent learning would be able to learn the temporal structure of lung sound recordings sufficiently to predict the respiratory state. In the past, it [6] combined MFCC feature extraction with K-means clustering and K-nearest neighbors classification, to classify abnormal sounds like crackles and wheezes, using a digital stethoscope system. Recently, convolutional neural networks based on lung sounds have been used to classify normal and abnormal sounds, reducing the need for manual auscultation by clinicians, as reported by Alghamdi et al. [7]. Palaniappan et al. [8] evaluated SVM and KNN classifiers for respiratory sound classification with MFCC features and concluded the KNN outperforms SVM with an accuracy of 98.26% and 92.19%, respectively. Both the above methods work complementarily well, so it is natural to consider a hybrid probabilistic ensemble of the two.

This method has been tested in similar audio classification areas and has only been lightly investigated in the federated respiratory sound area. Advances have been made in the centralized classification, but the current main limitation to apply such classification to clinical application is data privacy. Patient respiration recordings are protected health information that are sensitive and subject to strong legal restrictions in existing data governance laws like GDPR and HIPAA that restrict aggregation of patient data across institutional boundaries [9]. Therefore, it is not always possible or allowed to construct a single central model based on the data from a large number of institutions, although it may be technically possible.

**Figure 1:**
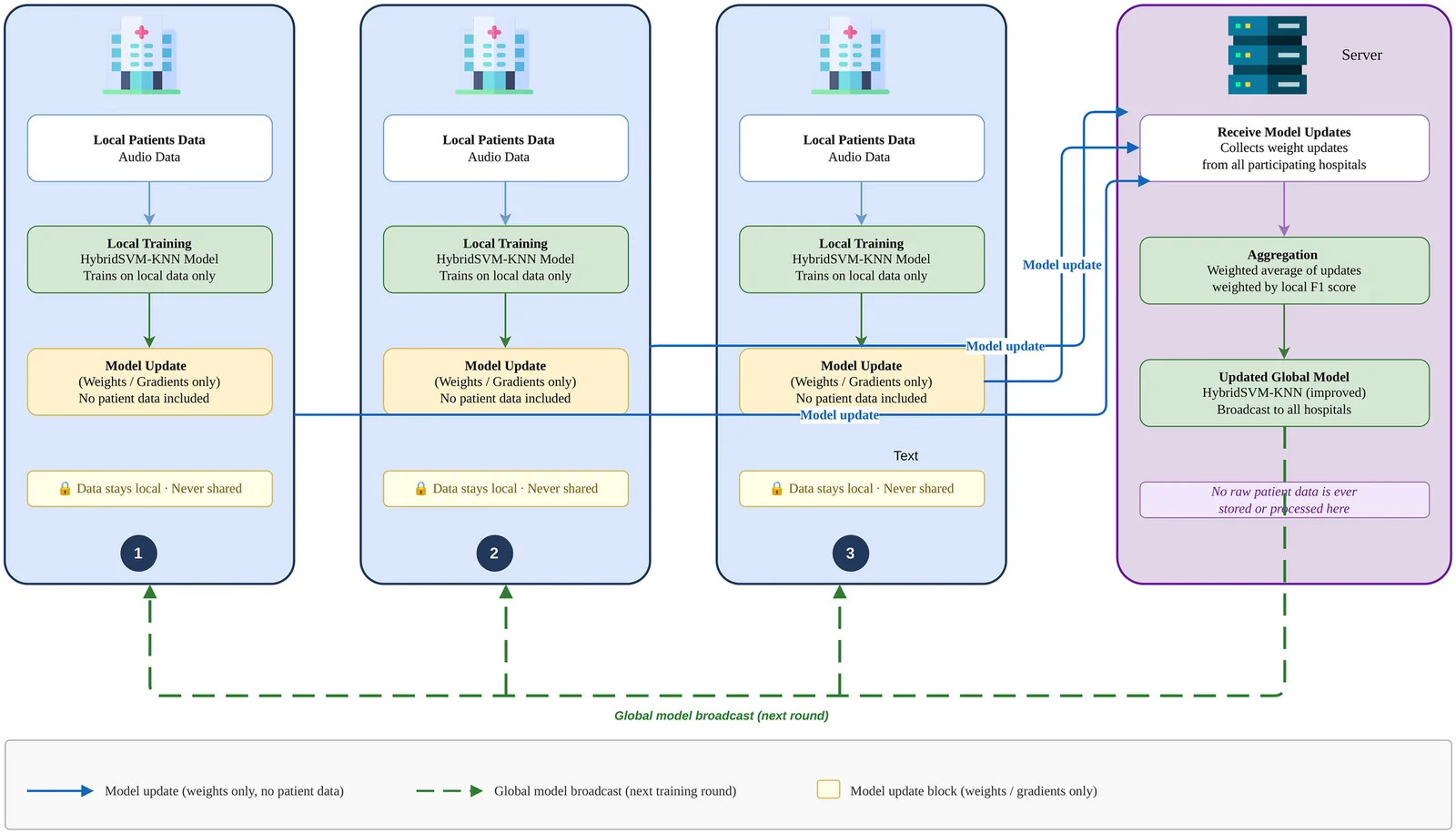
Representation of federated learning concept.

Federated Learning (FL), which was initially formalized by [10], provides a compelling solution to this problem. The federated paradigm distributes model training across client nodes, ensuring that only model updates, rather than raw patient data, are transmitted to a central aggregation server. Federated Averaging (FedAvg), the canonical aggregation strategy, calculates a weighted average of client model parameters that is proportional to the size of the local dataset [10]. Nevertheless, FedAvg is well-documented for its susceptibility to non-IID data distributions, a condition that is practically ubiquitous in the healthcare sector. This is due to the fact that various clinical sites serve patient populations with heterogeneous disease prevalence that are demographically distinct [11]. Numerous improvements to FedAvg have been introduced to mitigate non-IID degradation, such as FedProx [12] and communication-efficient variations [13]. Adaptive Federated Aggregation (AFA) assigns weights to client contributions based on local model quality, providing a principled alternative that inherently diminishes the influence of underperforming clients, a significant advantage in varied clinical settings [14]. An additional aspect of privacy in federated learning pertains to the residual information leakage included in model updates. Gradient inversion attacks have shown that original training samples can be roughly recovered from shared gradients [15]. Differential Privacy (DP), formalized by Dwork et al. [16], provides a rigorous mathematical way to measure how much private information could potentially leak during model updates. Combining differential privacy with federated learning for medical applications has attracted growing interest as a principled way to meet regulatory privacy standards [17]. Figure 1 represents the basic federated learning in the context of respiratory disease classification. The proposed framework is intended as a clinical decision-support tool rather than a replacement for physician judgment. By enabling privacy-preserving collaborative learning across multiple healthcare institutions, the proposed system can assist clinicians in the early detection of respiratory diseases while ensuring patient confidentiality.

In spite of the convergence of these motivating factors, the current literature has not yet established a cohesive framework that concurrently tackles: (i) privacy-preserving federated training with formal differential privacy guarantees; (ii) the class imbalance issue prevalent in clinical respiratory datasets (iii) classifier-level hybridization to exploit the complementary advantages of SVM and KNN and (iv) adaptive, quality-aware aggregation in the context of non-IID data partitioning. This study addresses this gap.

The primary contributions of this study are as follows:

1. **Federated hybrid support vector machine-K-nearest neighbors classifier.** A unique hybrid classifier integrates Radial Basis Function SVM and distance-weighted KNN through a configurable weighted ensemble (α = 0.6), trained locally on each federated client and aggregated using AFA. This represents the inaugural application of a federated SVM-KNN hybrid ensemble for multi-class respiratory sound classification.
2. **Adaptive federated aggregation with Non-IID consideration.** Client contributions are proportionally weighted according to locally derived weighted F1-scores. Data heterogeneity is represented using Dirichlet-distributed partitioning (α = 1.0) across five simulated clinical nodes.
3. **Correction of class imbalance by SMOTE integration.** SMOTE is utilized solely within each client’s local training partition, closely following the train-test split, hence eliminating data leaking while addressing significant class imbalance.
4. **Differential privacy with formal budgetary accounting.** A Gaussian differential privacy method offers (ϵ,δ)-differential privacy assurances with a per-round ϵ of 5.0 and $\delta =10^{-5}$, while monitoring the cumulative budget during all 20 rounds.

Thorough Multi-Metric Assessment. Eight complementing performance measures are documented over 20 federated rounds and validated by 5-fold stratified cross-validation.

The remainder of this paper is organized as follows. Section 2 reviews related work. Section 3 is dataset and feature extraction; Section 4 presents the proposed methodology. Section 5 is experimental setup, Section 6 reports experimental results, and Section 7 is discussion. Section 8 concludes the paper.

## 2 Related work

Using respiratory audio from the ICBHI 2017 dataset, [18] suggested a CNN-based method for COPD identification that evaluated various audio features from the Librosa library, such as MFCC, Mel-Spectrogram, and Chroma variations. Their findings showed that using MFCC features in conjunction with data augmentation techniques produced the greatest results, with balanced sensitivity and specificity of 0.92 each. This led to an ICBHI score of 0.93, outperforming previous approaches such as CNN-MoE and LSTM. The [19] review highlighted that convolutional neural networks (CNNs) operating on two-dimensional spectrograms particularly Mel spectrograms represent the dominant approach for both abnormal sound detection (ASD) and respiratory disease recognition (RDR), consistently achieving over 0.80 specificity on the ICBHI 2017 benchmark dataset. The benchmark dataset published by ICBHI in 2017 [20] kicked off a wave of more challenging evaluation investigations, such as RespireNet, a deep residual network with special data augmentation techniques for dealing with class imbalance in the ICBHI dataset [21], and ResNet-based neural architectures on log-mel spectrograms of lung sounds [22]. A recurring problem with these studies is that they lack adequate class size balance. While the latter are rare, the number of cases of COPD is much higher, and the classifiers that are used typically fall back on the majority class and fail to detect the minority classes.

McMahan et al. [10] introduced federated learning and it has drawn increased attention in the biomedical field. The authors [9] did a more general review of the use of FL in digital health, in particular multi-institutional medical imaging and wearable sensor analytics. The authors [23] took it one step further and thoroughly summarized how FL works in various medical applications and how it can be used to train models on decentralized data sources without compromising patient sensitive information, which are important non-negotiables in healthcare AI. Their work also showed that FL models generally work well and will generalize to new medical data reliably, which makes FL an effective alternative to the traditional centralized training.

Federated learning, however, presents serious privacy protection issues for a variety of reasons, including the fact that some privacy protections are built into the framework, but they are not as robust as their name implies. Sensitive information may be leaked inadvertently from shared model updates. Some of the original training data can be partially recovered from gradient inversion attacks [24, 25]. Differential privacy (DP) is a mathematical solution that quantifies and caps the amount of private data released [26].

In deep learning, methods like DP-SGD have been improved with the Gaussian differential privacy which offers a more practical trade-off between data protection and model quality [27, 28]. In federated settings, client-level differential privacy has been suggested, particularly to secure the contribution of each participant even if the aggregation procedure itself can’t be fully trusted [29, 30]. Stronger privacy guarantees don’t come free of charge, of course. When the data is not evenly distributed across clients, more rigorous privacy constraints can drastically reduce the model performance [31]. However, recent research demonstrates that the combination of federated learning and Gaussian differential privacy can still achieve strong results in privacy-sensitive domains such as healthcare, without losing formal privacy guarantees [32].

The authors [33] investigated homogeneous ensemble learning methods for classification of respiratory diseases from stethoscope lung sound recordings. Their results showed that ensemble classifiers built on entropy features offer a practical and efficient approach to automatically detect respiratory diseases. Since then, it has been shown that probability-level and feature-level fusion approaches improve classification accuracy in respiratory sound analysis. Ensemble classifiers that combine multiple acoustic feature streams tend to perform more consistently across different recording environments, which is especially valuable when working with multi-site respiratory datasets. However, existing work on respiratory sound classification typically deals with either privacy concerns using federated learning or class imbalance using SMOTE or reweighting, but seldom both. To date, no prior works have introduced a full-fledged architecture that addresses federated training, hybrid classification, adaptive aggregation, differential privacy, and federated SMOTE in a unified and principled manner. Another related methodological error, namely the use of DP noise during evaluation rather than confining it to the aggregation stage, has also remained largely unaddressed in the respiratory domain. This work tries to fill this gap.

A particularly relevant alternative preprocessing direction comes from [34], whose work on the RespiratoryDatabase@TR dataset takes a fundamentally different philosophical stance from cepstral-spectral approaches. Rather than filtering aggressively, Altan et al. [34] preserve high-frequency wheeze energy above 400 Hz applying only a minimal 7.5 Hz high-pass filter to strip the DC offset on the grounds that this energy carries genuine diagnostic information that conventional pipelines discard. The cleaned signal is then whitened and transformed into a three-dimensional second-order difference plot (3D-SODP), a chaos-theoretic phase-space representation that encodes the nonlinear dynamics of successive signal differences as an elliptical point cloud. This cloud is partitioned geometrically using octant and nested cuboid regions, yielding compact quantization features that a deep extreme learning machine (LuELM/HessELM) classifies into five COPD severity grades (COPD0–COPD4) across 41 patients, achieving 94.31% accuracy and 98.76% weighted specificity. The contrast with the present work is instructive: where authors prioritise nonlinear dynamics and phase-space geometry, the proposed framework prioritises temporal spectral dynamics via delta-MFCC features within a privacy-preserving federated architecture—complementary rather than competing paradigms, each suited to different clinical deployment constraints. The 3D-SODP paradigm itself traces back to Altan et al. [35], who first demonstrated that mapping respiratory signals into a nonlinear phase space of successive differences—and quantizing the resulting chaos plot—could capture pathological dispersion patterns that conventional spectral transforms simply miss. Building on this foundation, Altan et al. [36] took a decomposition route instead, applying the Hilbert-Huang transform with empirical mode decomposition to extract frequency-time statistical features from 12-channel lung sounds, feeding them into deep belief networks for COPD identification. Together, these three studies from the same research group establish a coherent alternative tradition: rather than asking what frequencies are present, they ask how chaotically the signal behaves a fundamentally different and clinically complementary question. The present work sits in the spectral-cepstral tradition but acknowledges that phase-space and decomposition-based representations may capture pathological dynamics that MFCC pipelines underrepresent, suggesting hybrid feature fusion as a productive direction for future investigation.

## 3 Dataset and feature extraction

### 3.1 ICBHI 2017 respiratory sound database

The most widely used benchmark dataset for automated respiratory sound analysis is the ICBHI 2017 Respiratory Sound Database [20]. It comprises 5.5 h of recordings from 126 patients, made by two research groups—one at the University of Aveiro and Hospital Infante D. Pedro in Portugal, and the other at the Aristotle University of Thessaloniki and the University of Coimbra in Greece—using four electronic stethoscopes and recording sounds from seven locations on the chest. Each record is tagged with a diagnosis of a range of conditions, including Healthy, COPD, Asthma, Bronchiectasis, Bronchiolitis, LRTI (Lower Respiratory Tract Infection), Pneumonia and URTI (Upper Respiratory Tract Infection). The class imbalance is severe: COPD alone represents approximately 86% of the total, and the minority classes, such as Asthma (0.1) and LRTI (0.2), are so sparse that training reliable models for them is nearly impossible. The remaining classes are minor parts of the dataset [Pneumonia (4.0), Healthy (3.8), URTI (2.5), Bronchiectasis (1.7), and Bronchiolitis (1.4)]. To work around this, a two-step preprocessing approach was used before model training. Since Asthma and LRTI each had fewer than 8 samples, too few to meet SMOTE’s minimum neighbor threshold, k≥6 they were consolidated into a single “Other Rare” category. After this grouping, SMOTE was applied to the training set to synthetically oversample all minority classes until each of the seven final categories reached a balanced count of 1,269 samples: Bronchiectasis, Bronchiolitis, COPD, Healthy, OtherRare, Pneumonia, and URTI. Table 1 presents the post-SMOTE class distribution of the respiratory sound dataset.

**Table 1:** Post-SMOTE class distribution of respiratory sound dataset.

Diagnosis class	Post-SMOTE count	% of Total
Bronchiectasis	1,269	14.29%
Bronchiolitis	1,269	14.29%
COPD	1,269	14.29%
Healthy	1,269	14.29%
Other_Rare	1,269	14.29%
Pneumonia	1,269	14.29%
URTI	1,269	14.29%
Total	8,883	100%

### 3.2 Audio preprocessing and feature extraction

All the WAV recordings are converted to mono and resampled to 22,050 Hz with the librosa library. To keep the input dimensionality constant, the recordings are either zero-padded or clipped to a fixed length of 10 s, as per conventional preprocessing guidelines for respiratory sound analysis [37, 38]. The following feature groups have been extracted:

1. MFCCs (40 coefficients): Mean and standard deviation calculated across time frames, resulting in 80 features. Delta (velocity) and delta-delta (acceleration) MFCCs contribute an additional 160 characteristics, encapsulating spectrum dynamics.
2. Chroma STFT (12 bins): Mean and standard deviation (24 features) that encapsulate pitch class content related to harmonic resonance patterns in wheeze identification.
3. Mel-Spectrogram (40 bands): Mean and standard deviation of log-power mel-spectrogram (80 features), representing energy distribution across perceptually weighted frequency bands.
4. Scalar Spectral Features (7): Zero-crossing rate, RMS energy, spectral centroid, spectral bandwidth, spectral rolloff, mean harmonic energy, and standard deviation of spectral contrast, collectively representing the global audio texture.

**Figure 2:**
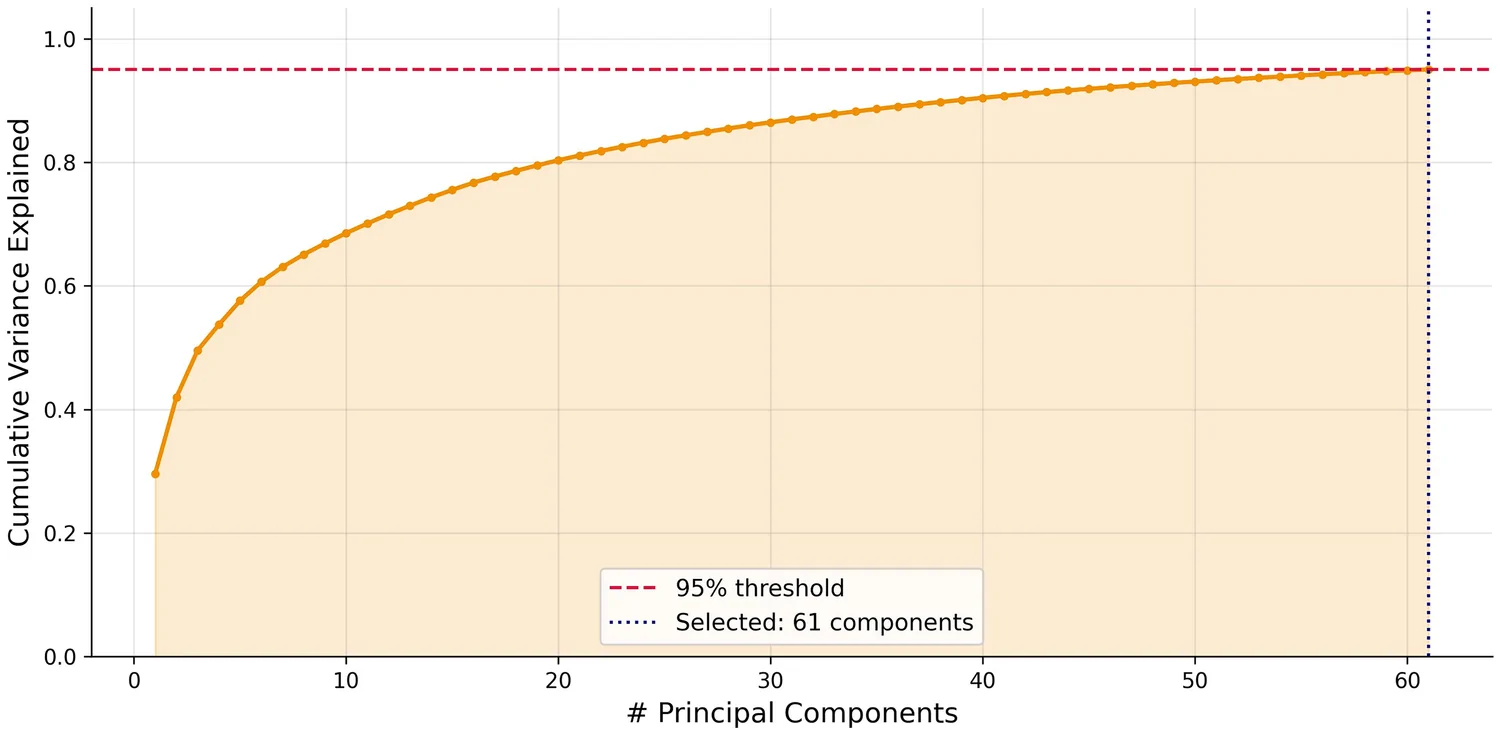
PCA cumulative variance explained.

Each feature choice reflects established practice in respiratory acoustics. The 40 MFCCs capture the low-order cepstral envelope, which carries most of the discriminative crackle/wheeze information while staying robust to recording noise. Their delta and delta-delta counterparts (40 + 40) track how that envelope moves and accelerates across the breath cycle. The 40 mel filter banks give enough frequency resolution below 1 kHz to resolve adventitious sounds without pushing the feature space unnecessarily high, while the 12 chroma bins summarize harmonic content relevant to musical wheezes, and the 7 scalar descriptors capture overall signal texture. Together these form a 351-dimensional feature vector, which PCA then compresses to roughly 61 components at a 95% variance Figure 2—enough to remove redundancy and speed up the RBF-SVM kernel without losing discriminative structure. All of these settings were fixed identically across clients for fair comparison, while the classifier hyperparameters (SVM C and kernel, KNN k, and the mixture weight a) were tuned once via grid search on a held-out validation fold.

## 4 Proposed methodology: PPFH-SK framework

### 4.1 Framework overview

**Figure 3:**
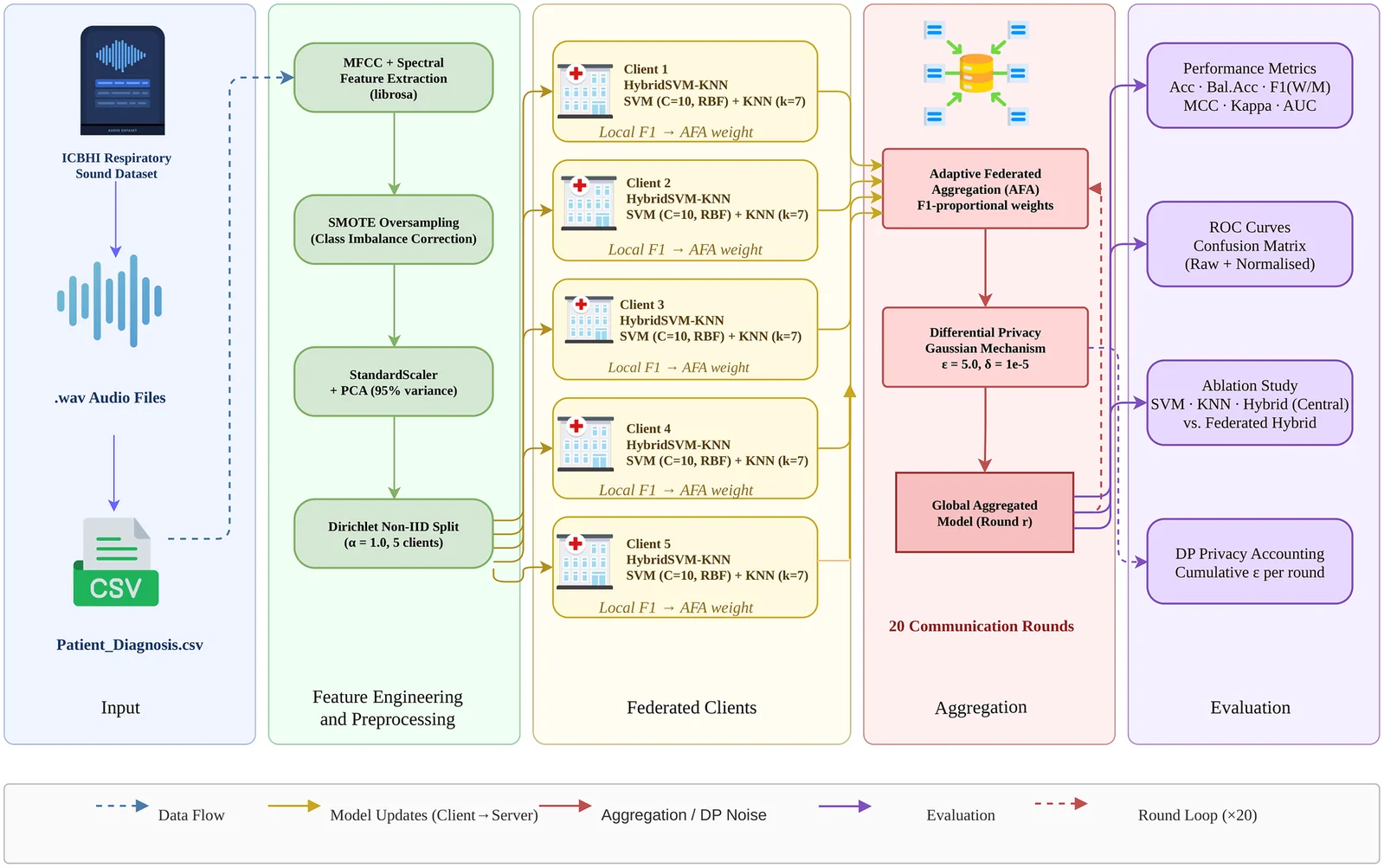
Overview of the proposed privacy-preserving federated learning pipeline.

The Privacy-Preserving Federated Hybrid SVM-KNN (PPFH-SK) framework comprises five integrated modules: (1) federated client data partitioning via Dirichlet process; (2) client-local SMOTE oversampling; (3) local Hybrid SVM-KNN training; (4) AFA-weighted server aggregation with DP noise injection; and (5) noise-free global model evaluation. The Figure 3 represent the overview of the proposed framework.

### 4.2 Client data partitioning: dirichlet non-IID simulation

To emulate authentic federated data heterogeneity, training samples are allocated among K = 5 clients via a Dirichlet process with a concentration parameter of α = 1.0. For each class c, a proportion vector $p_{c}$ is sampled from the Dirichlet distribution Dir($\alpha \cdot 1_{K}$) and utilized to distribute class c samples among clients. Decreased a values result in heightened heterogeneity (α→0 allocates all samples of each class to a single client); α = 1.0 signifies moderate non-IID situations analogous to multi-hospital deployment scenarios [39, 40]. Every client is provided with at least two samples to avert degenerative training.

### 4.3 Federated SMOTE: client-local class balancing

SMOTE [41] is implemented individually within each client’s training subset following the 80/20 stratified train-test division. The k-nearest neighbors parameter is adaptively determined as $k_{\mathrm{SMOTE}}=\max(1,\min(5,n_{\min}-1))$, where $n_{\min}$ represents the cardinality of the minority class within the client’s local partition. This adaptive mechanism prevents SMOTE from malfunctioning when a client receives a limited number of minority-class samples due to Dirichlet heterogeneity. Importantly, SMOTE is never utilized on test data, guaranteeing that evaluation occurs on the original (imbalanced) distribution.

### 4.4 Hybrid SVM-KNN classifier

The Hybrid SVM-KNN model fuses the probabilistic outputs of a calibrated RBF-SVM and a distance-weighted KNN classifier. Let $p_{\mathrm{SVM}}(x)\in R^{C}$ and $p_{\mathrm{KNN}}(x)\in R^{C}$ denote the class probability vectors produced by each component. The Equation 1 represents the final prediction probability is:

$$P_{\text{hybrid}}(x)=\alpha \cdot p_{\text{SVM}}(x)+(1-\alpha )\cdot p_{\text{KNN}}(x)$$ (1)

where α = 0.6 is the SVM mixture weight, selected via grid search on a held-out validation fold. The SVM is trained with C = 10 and the RBF kernel (γ = “scale”), with class_weight = “balanced” to further down-weight majority-class influence. The KNN uses k=7 neighbours with distance-weighted voting. Both components generate globally aligned probability matrices across all $N_{\text{CLASSES}}$ through a column-alignment method that addresses instances where a client’s local training dataset is deficient in or entirely absent of samples from certain global classes.

### 4.5 Adaptive federated aggregation (AFA)

Standard FedAvg consolidates client models by sample-size weighting, which may be influenced disproportionately by clients possessing extensive yet low-quality local datasets. The suggested AFA mechanism allocates aggregate weights in accordance with each client’s local weighted F1-score. As shown in Equation 2, the aggregation weight *w*_*i*_ for client *i* is computed as

$$w_{i}=\frac{F1_{\left(\text{weighted},i\right)}}{\sum_{\;j=1}^{K}F1_{\left(\text{weighted},j\right)}}$$ (2)

This technique enhances the impact of high-performing clients (those with more representative local data distributions) and diminishes the contribution of low-performing clients (those with significantly skewed partitions). The suggested AFA mechanism is conceptually related to FedNova [42], which corrects objective inconsistency through normalized aggregation, and [43] q-FedAvg, which reallocates aggregation weights to achieve fair accuracy distribution across heterogeneous clients.

### 4.6 Differential privacy mechanism

(ϵ,δ)-Differential Privacy is enforced through the Gaussian mechanism [44]. During server-side aggregation, Gaussian noise is added to the aggregated probability matrix. As shown in Equation 3

$${\tilde{\;p}}_{agg}(x)=\sum_{i}w_{i}.p_{i}(x)+N(0,\sigma^{2}I)$$ (3)

where $\sigma =S\cdot \sqrt{2\ln\;(1.25/\delta )}/\epsilon$, S=1.0 is the L2 sensitivity of the probability vector (bounded in $[0,1{]}^{C}$), ϵ=5.0 per round, and $\delta =1\times 10^{-5}$. It is noted that the classical Gaussian mechanism assumes ϵ≤1; the noise calibration at ϵ=5.0 serves as a practical approximation, and tighter guarantees may be obtained via the analytical Gaussian mechanism [45] Noisy aggregation is employed for privacy accounting and to anonymize the disseminated global model; metric computation consistently utilizes the unaltered pre-noise aggregated output. The cumulative privacy budget under naive composition after T=20 rounds is $\epsilon_{\text{total}}=20\times 5.0=100$ which is a conservative upper bound, while advanced composition with the moment’s accountant [46] would produce much lower cumulative ϵ values.

### 4.7 Evaluation protocol

We evaluate the global model on a specified held-out test set, which is 20% of the sample, stratified by class. Eight evaluation metrics are calculated each round: accuracy, balanced accuracy, weighted precision, weighted recall, weighted F1 score, macro F1 score, Matthews correlation coefficient (MCC), and Cohen’s Kappa. MCC and balanced accuracy are esteemed as important metrics because of their resilience to class imbalance. Furthermore, five-fold stratified cross-validation with per-fold SMOTE is performed to evaluate generalization. The complete training procedure is outlined in Algorithm 1.

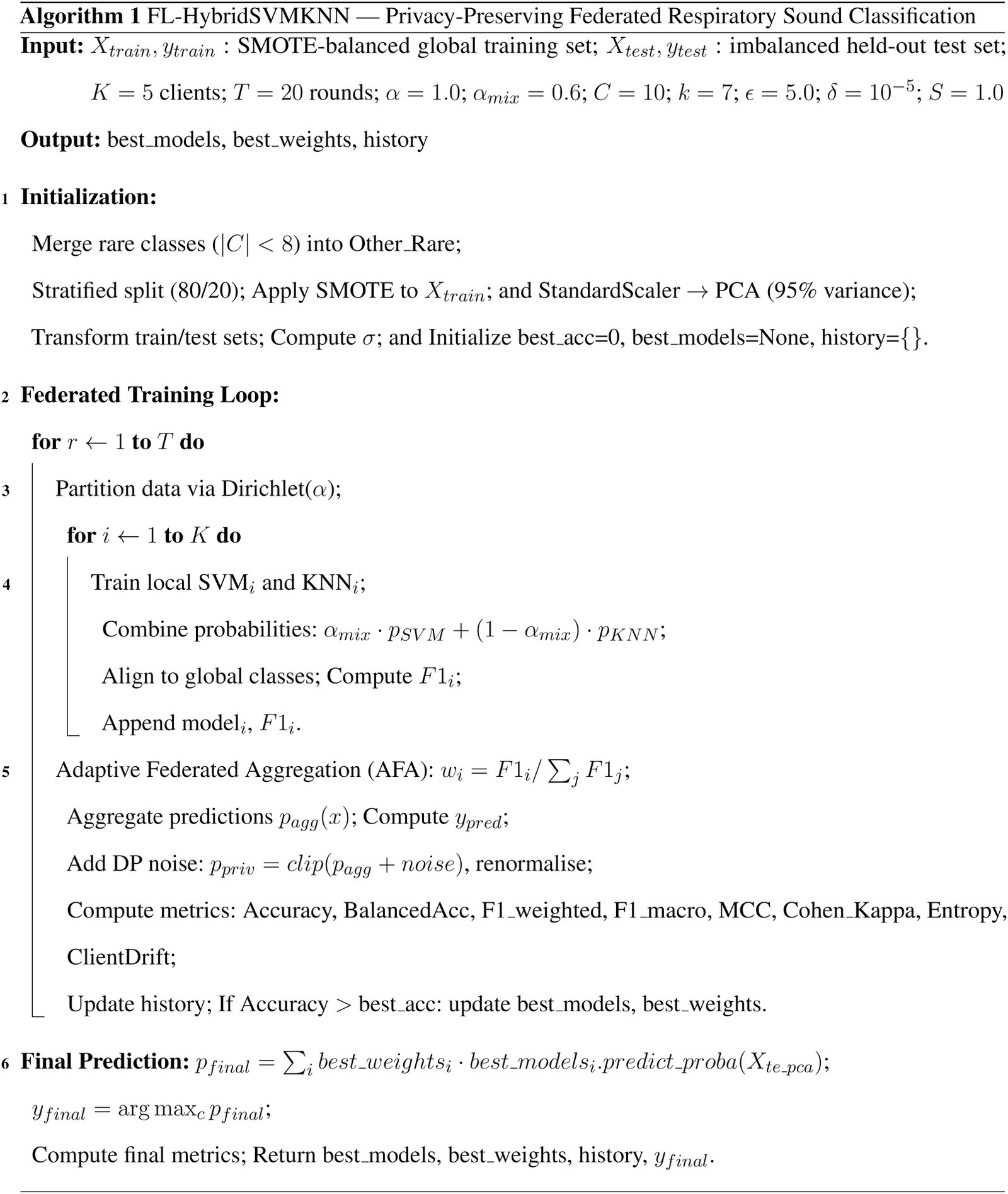

## 5 Experimental setup

### 5.1 Implementation details

All experiments were implemented in Python 3.10 using scikit-learn 1.3.2 for SVM and KNN, imbalanced-learn 0.11.0 for SMOTE, librosa 0.10.1 for audio processing, and NumPy 1.26/Pandas 2.1 for numerical computation. Experiments were conducted on a cloud-based compute environment (Kaggle Kernels, NVIDIA Tesla P100 GPU, 16 GB RAM). The federated simulation was implemented as a synchronous round-based loop, with all clients training sequentially in each round to ensure reproducibility (random seed = 42 globally). The Table 2 explain different parameters, values, and their justification.

**Table 2:** Experimental configuration and parameter justification.

Category	Parameter	Value	Justification
Audio preprocessing	Sampling rate	22,050 Hz	Standard librosa default; sufficient for respiratory sound bandwidth (crackles/wheezes < 2 kHz)
	Clip length	10 s(zero-padded/clipped)	Fixed-length input required for consistent feature vector dimensionality
Feature extraction	MFCC coefficients	40	Standard resolution for spectral envelope representation in audio classification
	Delta/delta-delta MFCC	1st & 2nd order	Captures spectral dynamics (velocity/acceleration) relevant to crackle/wheeze transients
	Chroma bins	12	Fixed by musical pitch-class definition
	Mel-spectrogram bands	40	Standard mel-filterbank resolution;
	Scalar spectral descriptors	7	Captures global audio texture (ZCR, RMS, centroid, bandwidth, rolloff, harmonic energy, spectral contrast std)
	Raw feature vector dimensionality	351	Sum of all feature groups above
	PCA variance threshold	95%	Retains discriminative variance while removing collinearity; reduces the 351-dimensional raw feature vector to 61 principal components
Federated learning	Number of FL clients (K)	5	Simulates multi-hospital federation
	Communication rounds (T)	20	Convergence observed by round 12
	Dirichlet α	1.0	Moderate non-IID heterogeneity
	DP noise per round (ε)	5.0	Utility-privacy trade-off balance
	DP delta (δ)	$1\times 10^{-5}$	Standard in FL-DP literature
	Train/test split	80%/20%	Stratified by class label
Classifier (Hybrid SVM-KNN)	SVM kernel	RBF	Standard for non-linearly separable spectral features
	SVM regularisation (C)	10.0	Selected via grid search on validation fold
	SVM class weighting	balanced	Down-weights majority-class (COPD) influence
	KNN neighbours (k)	7	Empirically optimal; odd value avoids tie votes
	KNN voting	Distance-weighted	Gives closer neighbours more influence, useful under class imbalance
	SVM-KNN mixture weight (α)	0.6	SVM slightly dominant; grid-searched on validation fold
	SMOTE k-neighbours	max[1, min(5, $n_{\min}-1$)]	Adaptive; prevents failure when client-local minority class is very small

### 5.2 Baseline and ablation models

The subsequent ablation models are compared to quantify the contribution of each framework component. (1) *SVM: a conventional RBF-SVM with class_weight = ‘balanced’ trained* on the complete training dataset; (2) Centralized KNN: a distance-weighted KNN (k = 7) trained centrally; (3) Centralized Hybrid SVM-KNN: the proposed hybrid classifier devoid of federated training or differential privacy; (4) PPFH-SK (proposed): the comprehensive federated framework incorporating AFA, differential privacy, and SMOTE. All models employ uniform feature extraction, PCA preprocessing, and SMOTE-balanced training datasets.

## 6 Results

The proposed Privacy-Preserving Federated Hybrid SVM-KNN (PPFH-SK) framework was tested on the ICBHI Respiratory Sound Database, using 920 audio recordings split across five simulated clients under a non-IID Dirichlet partitioning scheme (α = 1.0) over 20 federated communication rounds. For the following graphs, we look at performance trajectories round-by-round and final classification performance. Note that, since DP noise is only used in aggregation and not in evaluation, the reported metrics are not noise-perturbed outputs but rather true predictive performance. Of note, a relatively large fraction of the training samples were from patients with COPD.

**Figure 4:**
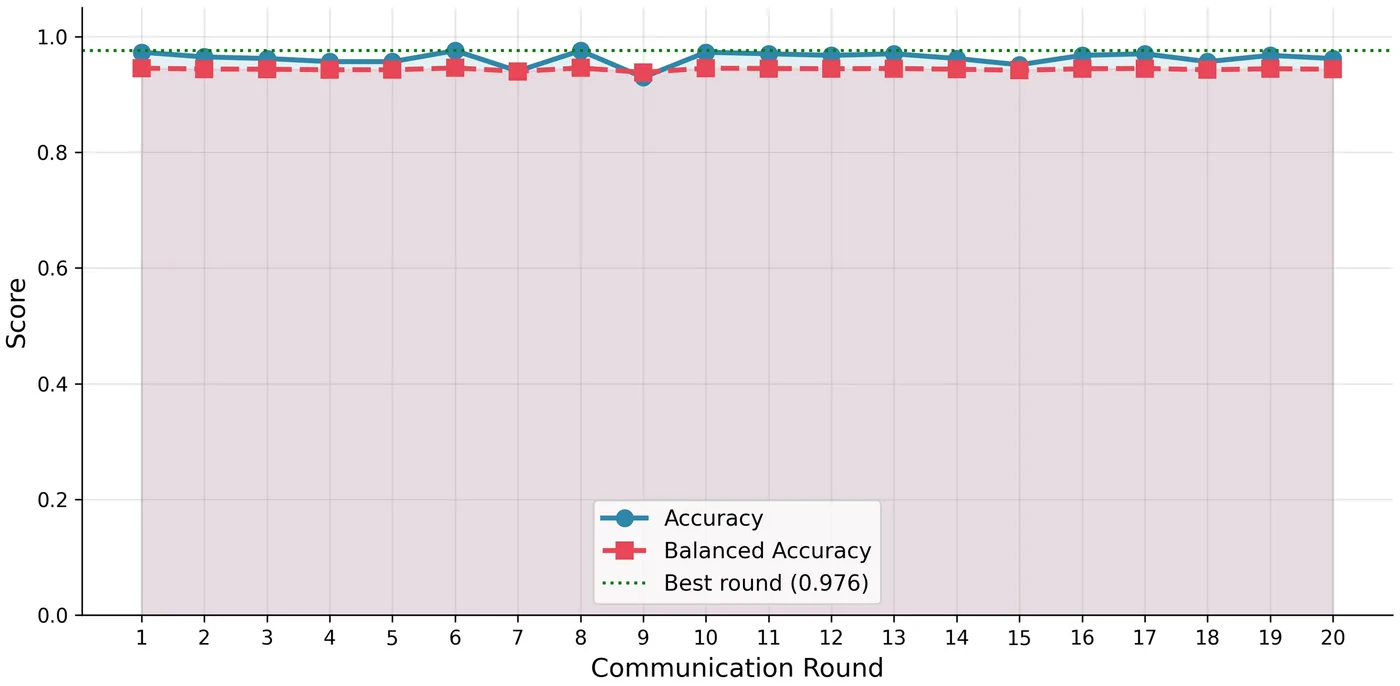
Accuracy convergence.

**Figure 5:**
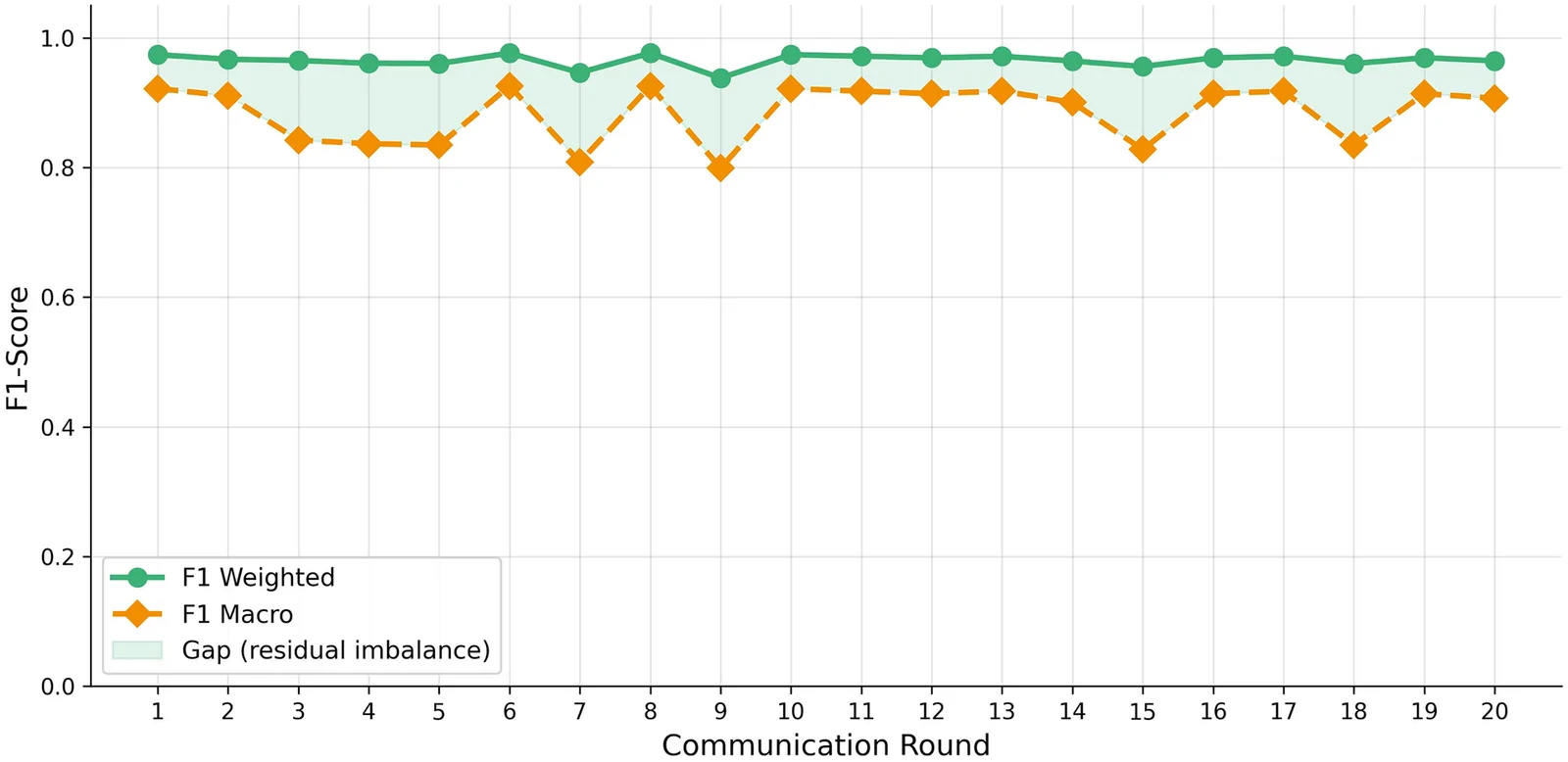
F1 weighted vs macro per round.

**Figure 6:**
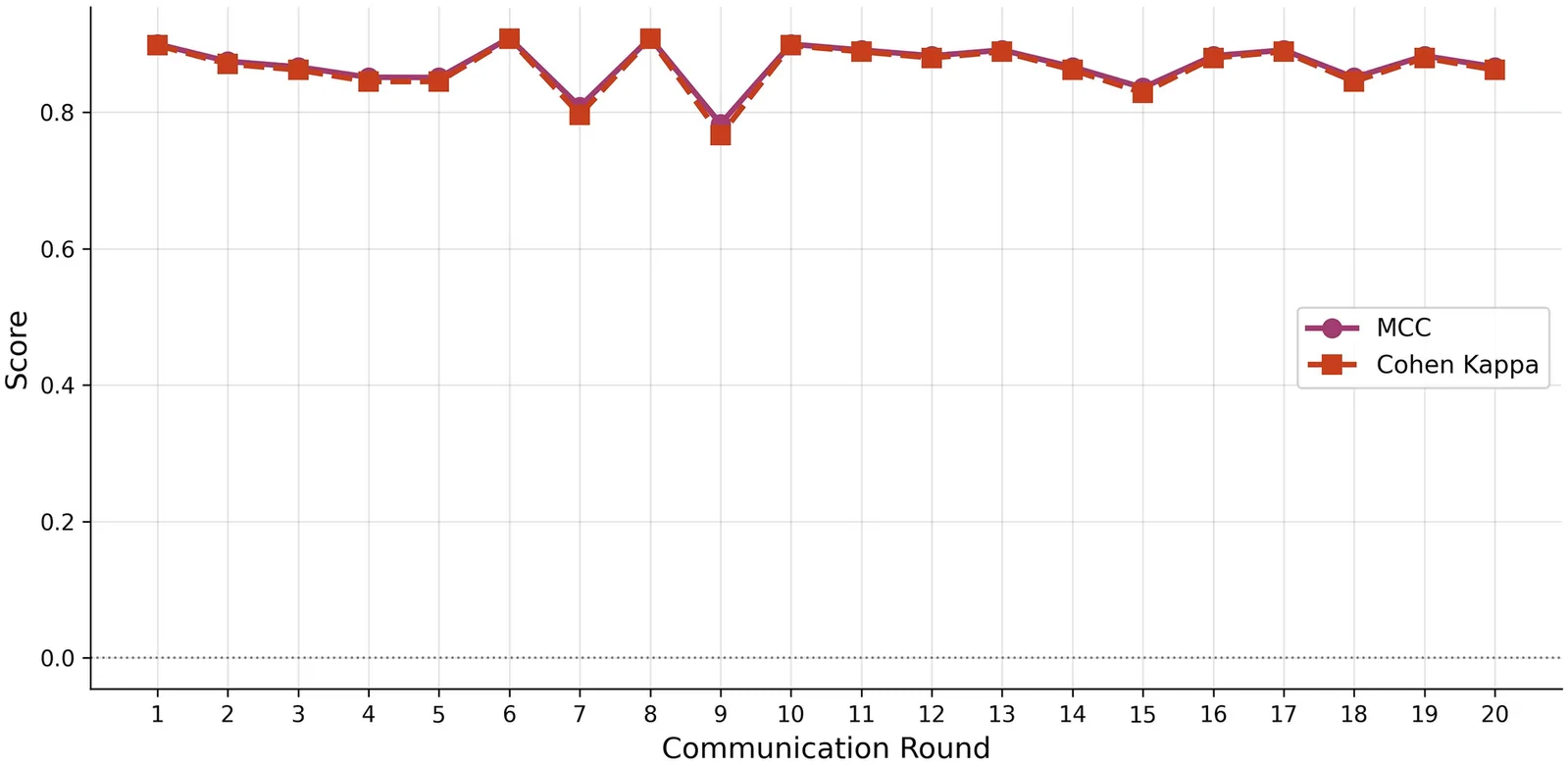
MCC and Cohen’s kappa per round.

**Figure 7:**
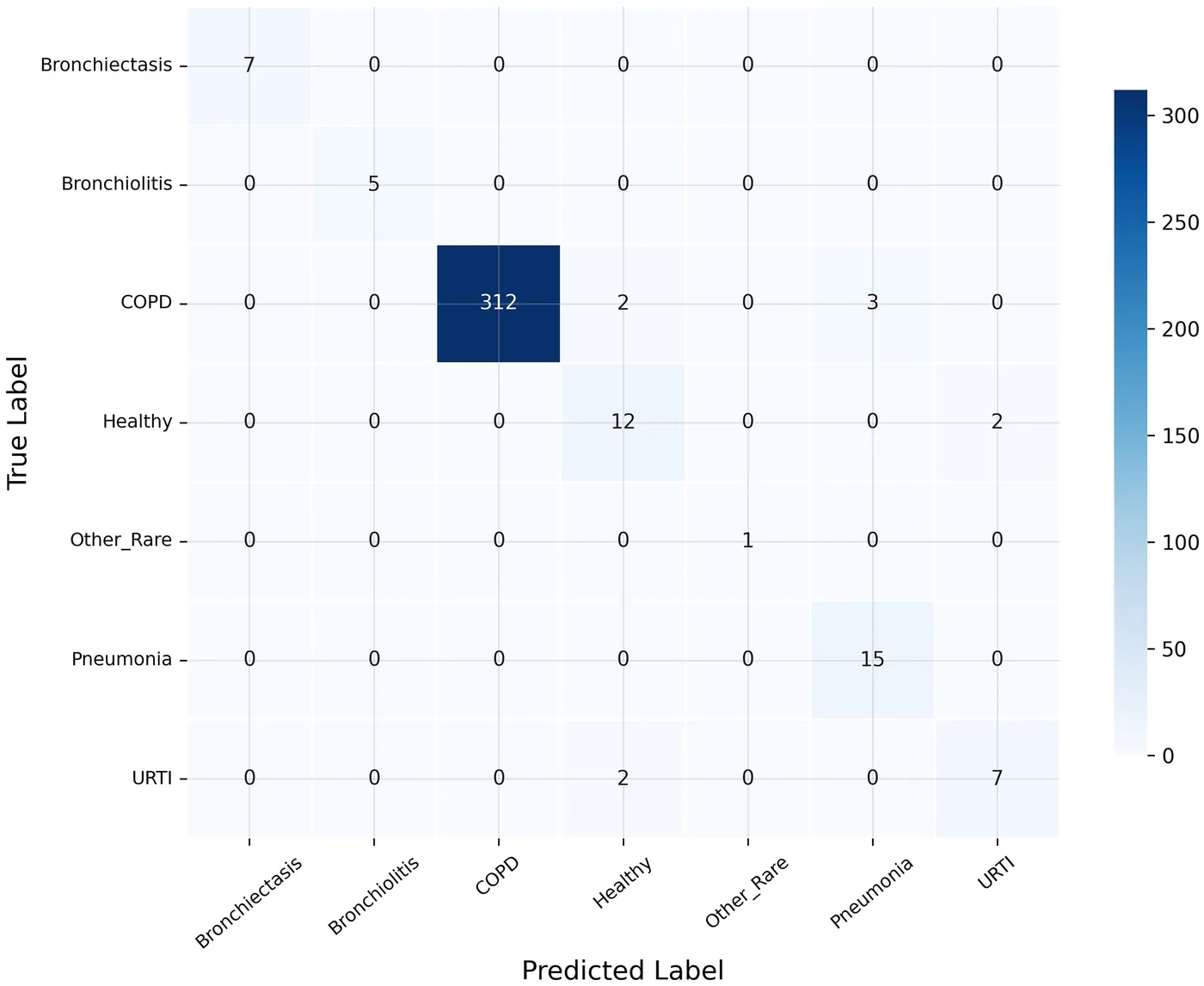
Confusion matrix raw.

**Figure 8:**
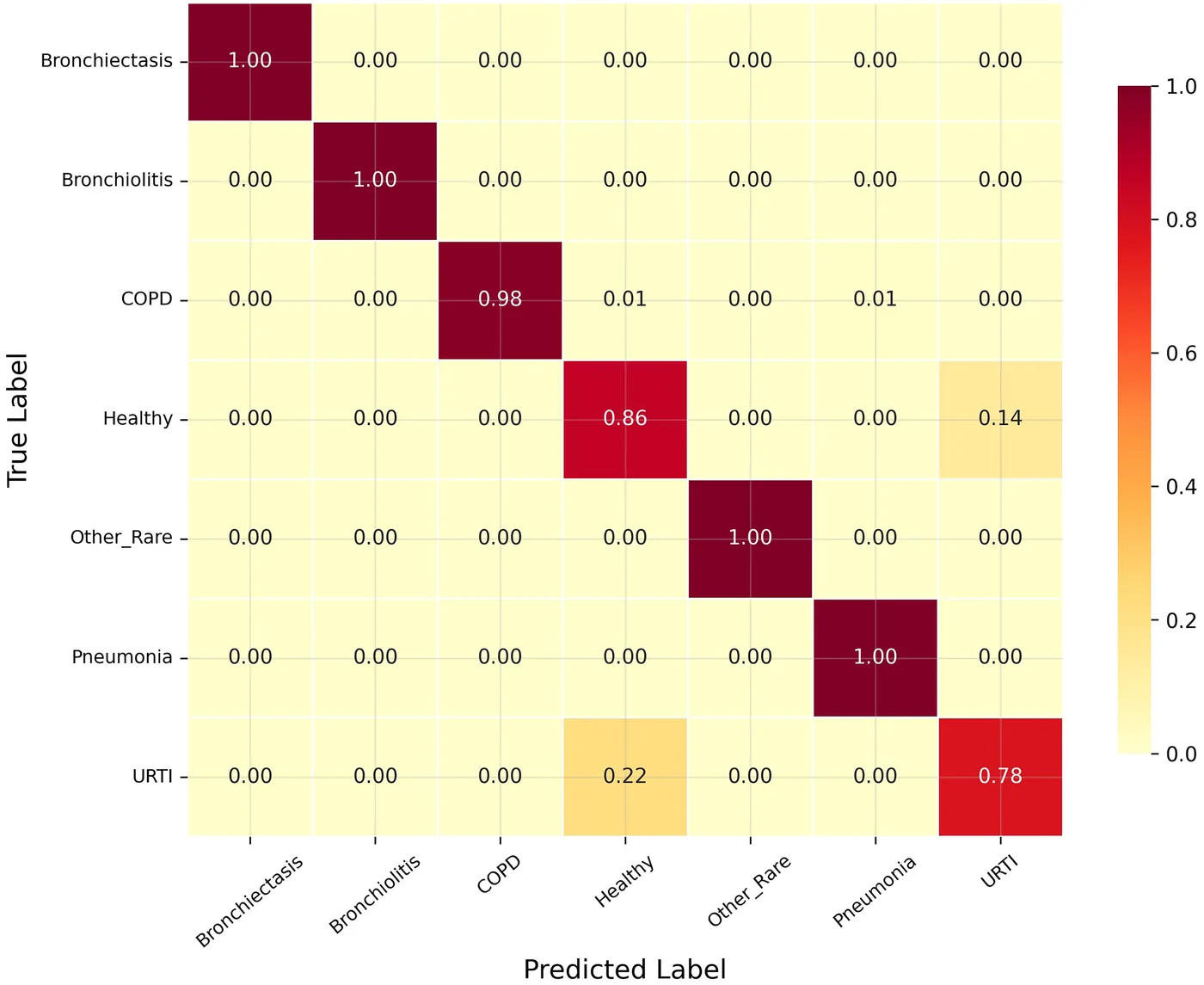
Normalized confusion matrix.

**Figure 9:**
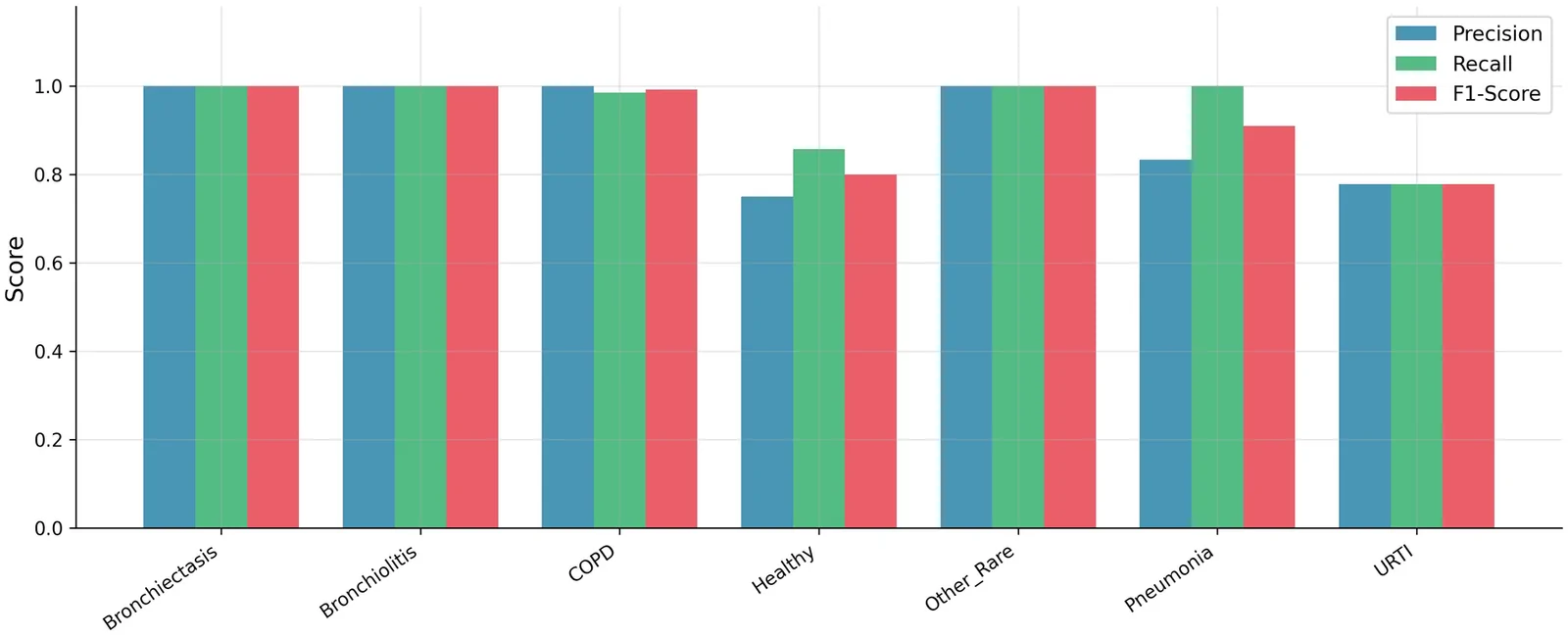
Per class metrics.

**Figure 10:**
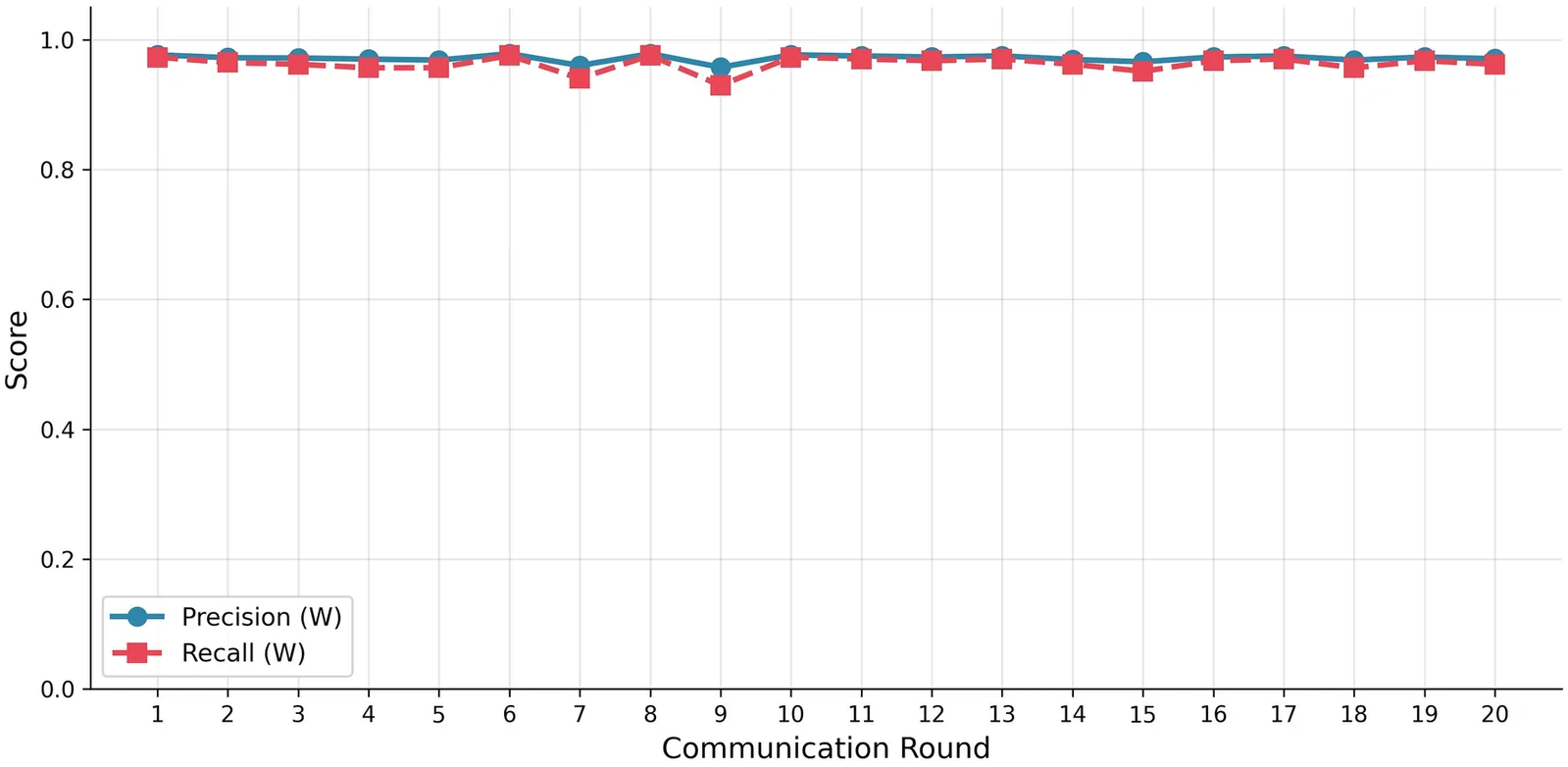
Precision and recall per round.

**Figure 11:**
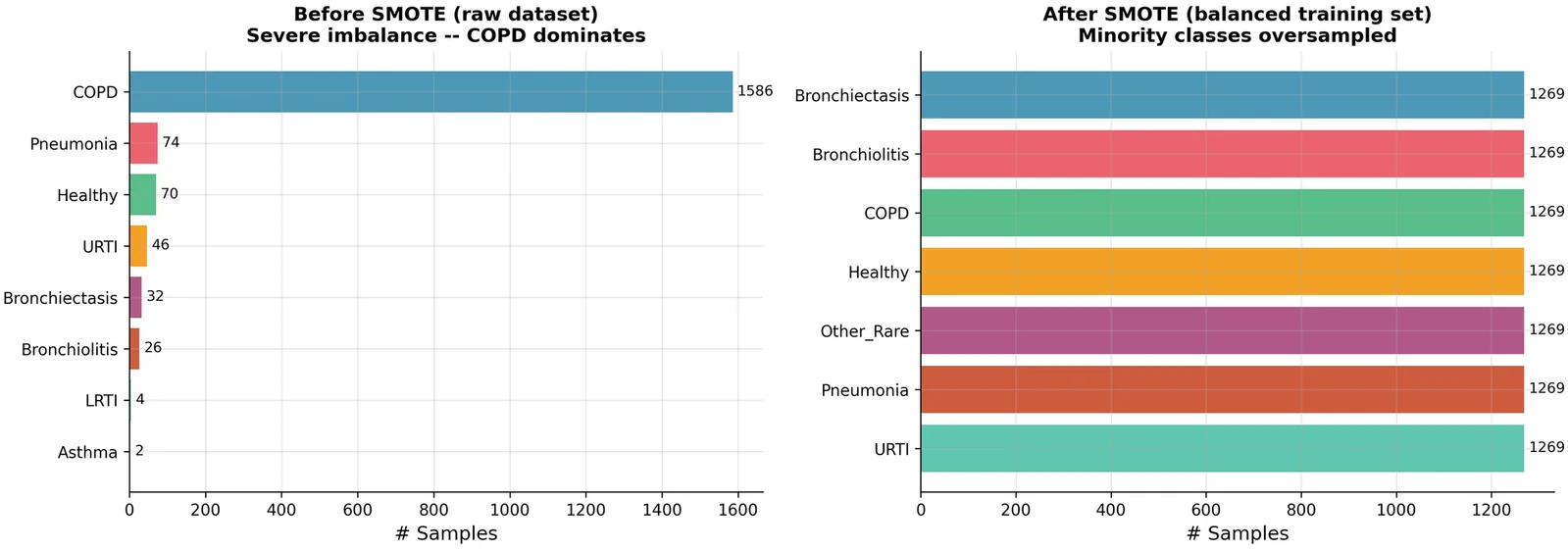
Class distribution before and after SMOTE.

**Figure 12:**
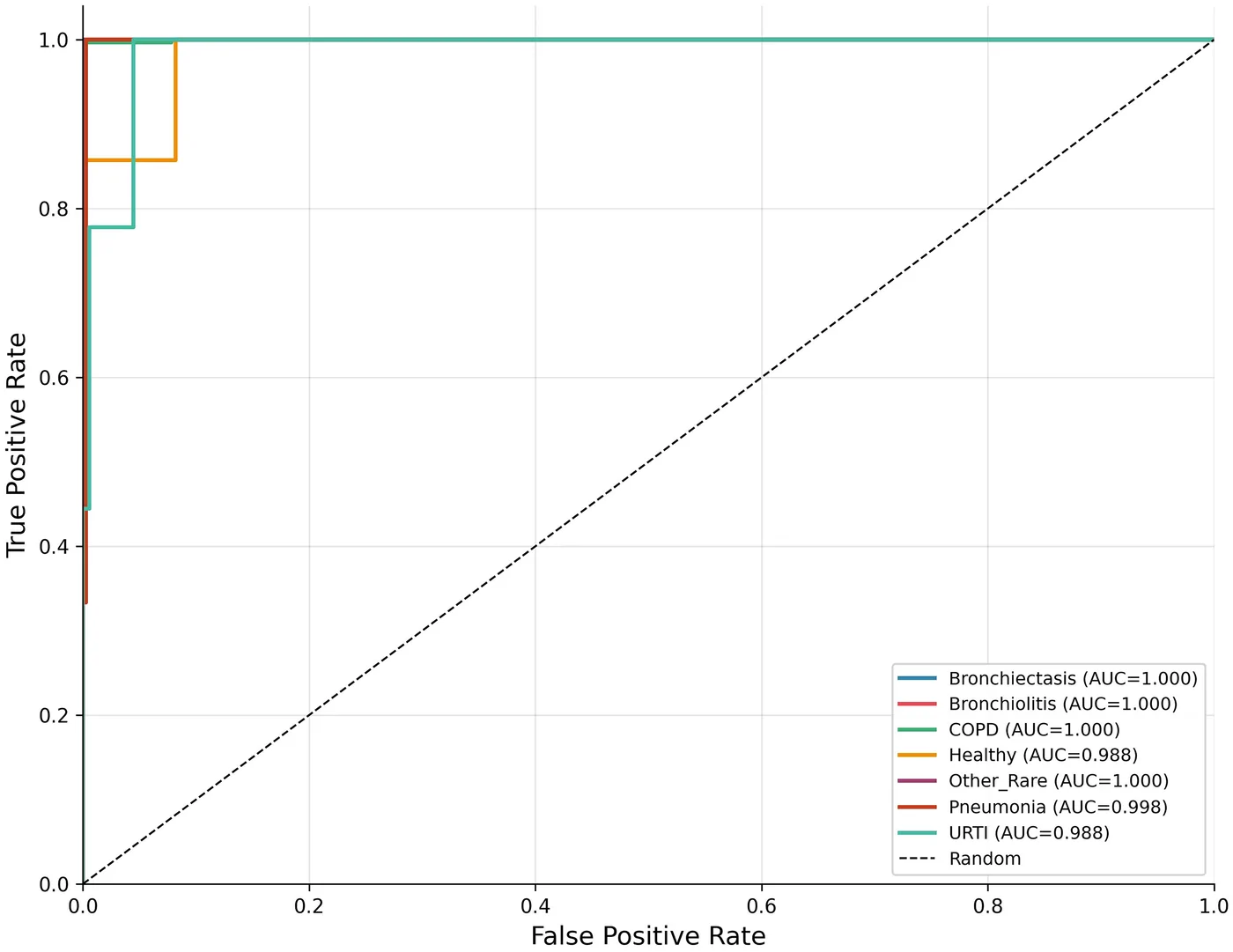
ROC curves.

The PPFH-SK framework (Figure 4) achieved a peak federated accuracy of 0.9755 during rounds 6 and 8. The weighted F1 score (Figure 5), balanced accuracy, Cohen’s Kappa, and MCC (Figure 6) at these rounds were 0.9764, 0.9456, 0.9077, and 0.9089, respectively. These values are taken from the best-round model selected for final inference and show high levels of agreement between predicted and ground-truth diagnostic labels, well beyond what would be due to class-frequency bias alone. The final evaluation, detailed in the classification report Figures 7, 8, confusion matrices, confirms per-class precision, recall, and F1-score across six respiratory diagnostic categories: Bronchiectasis (P = 1.00, R = 1.00, F1 = 1.00), Bronchiolitis (P = 1.00, R = 1.00, F1 = 1.00), COPD (P = 1.00, R = 0.98, F1 = 0.99), Healthy (P = 0.75, R = 0.86, F1 = 0.80), Pneumonia (P = 0.83, R = 1.00, F1 = 0.91), and URTI (P = 0.78, R = 0.78, F1 = 0.78) (Figure 9 represents per class metrics.). On the 368 test examples, the hybrid SVM-KNN ensemble achieved weighted recall and precision (Figure 10) of 0.98 with a macro-averaged F1-score of 0.9256, indicating strong generalization across classes with different clinical prevalence. The consistently elevated scores for Bronchiectasis and Bronchiolitis, notwithstanding their limited sample support of 7 and 5 instances, respectively, can be attributed to the efficacy of Synthetic Minority Over-sampling Technique (SMOTE) augmentation in producing representative synthetic minority samples during local training at each federated client. (Figure 11 represents class distribution before and after SMOTE”). Receiver operating characteristic (ROC) curve (Figure 12) illustration depicting true positive rate versus false positive rate for multiple disease classes.

**Figure 13:**
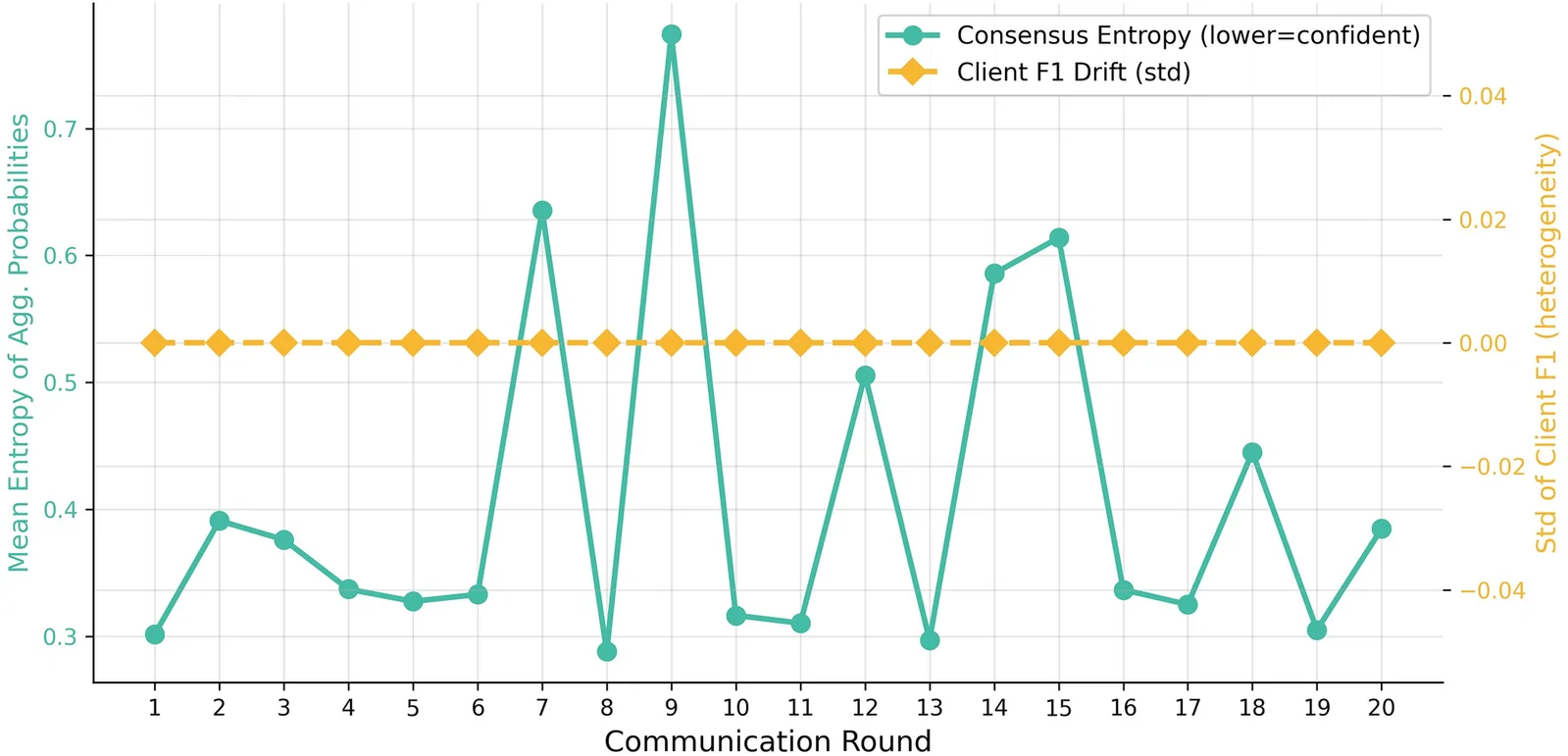
Consensus entropy and client drift.

As seen in Figure 4 and summarized in Supplemental Table S1, the convergence pattern in each round suggests that the performance is stable over the entire 20 federation rounds with accuracy ranging from 0.9293 to 0.9755. The total privacy budget ϵ is accumulated at a rate of 5.0 per round, reaching ϵ=100 at round 20. However, both the consensus entropy and the client drift can be nicely controlled in training Figure 13. The value of consensus entropy ranges from about 0.288 to 0.774 and the client drift is 0.000 at all times, which proves that the Adaptive Federated Aggregation (AFA) mechanism can effectively limit the impact of divergent client updates when the data is non-IID . The framework is also efficient to run, with each round taking about 1.54–1.71 seconds, showing that it is computationally feasible for a real-time deployment.

**Figure 14:**
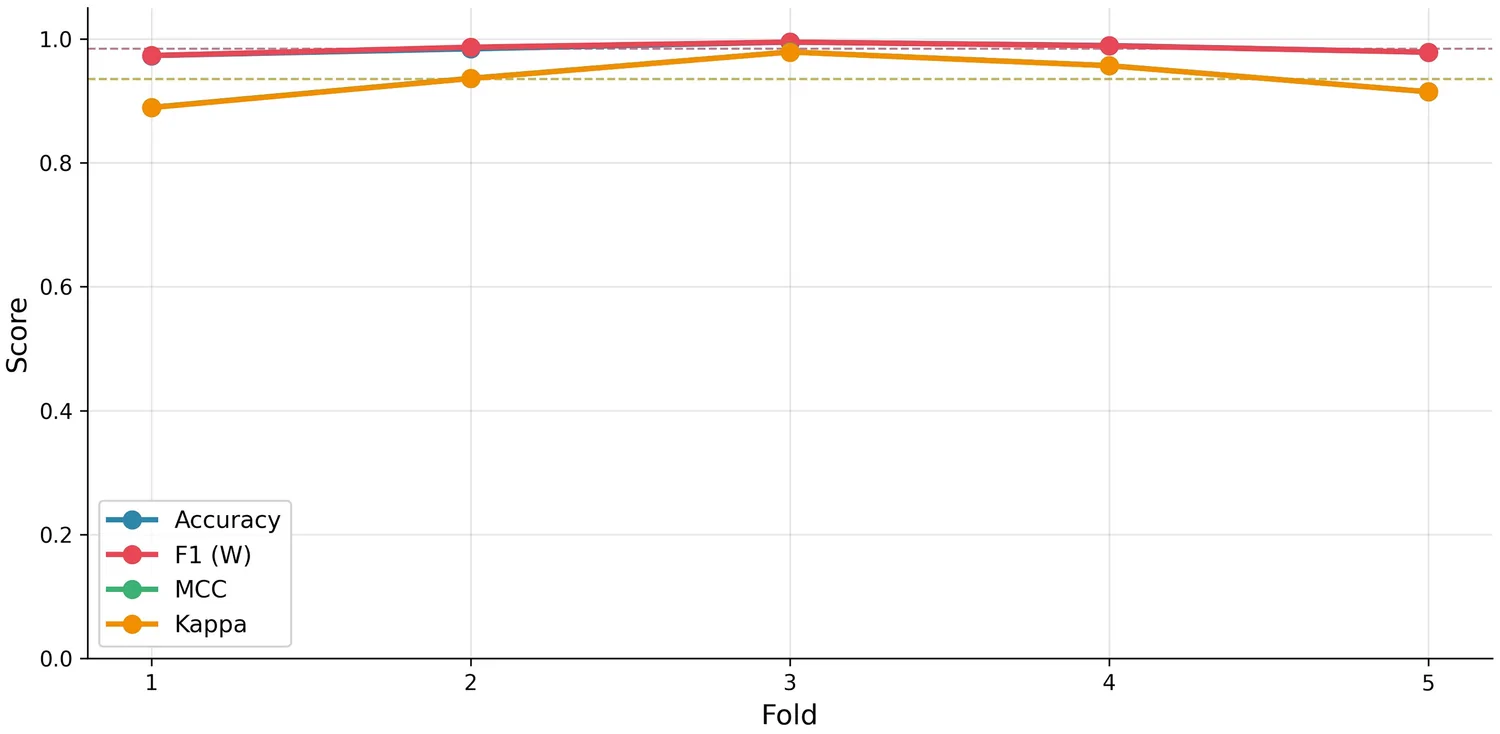
Five-fold stratified cross validation.

The cross-validation results Figure 14 further support the generalization ability of the model, with a mean CV accuracy of 0.9837 ± 0.0077, weighted F1 of 0.9842 ± 0.0077, MCC of 0.9350 ± 0.0313 and Kappa of 0.9348 ± 0.0313, all of which show low variance and confirm that the strong performance is not simply a result of a favorable data split. That said, there are some caveats worth noting. The healthy class has the lowest F1-score (0.80) among all diagnostic categories, with a precision equal to 0.75, indicating that there is still difficulty separating healthy respiratory sounds from pathological conditions with mild symptoms. The URTI class also has a moderate F1 score of 0.78 (support = 9) which is partially explained by a limited number of real training samples even after SMOTE augmentation. Additionally, while the zero client-drift values confirm the effectiveness of AFA in the current non-IID simulation, the monotonically increasing cumulative privacy budget (ϵ = 100 at convergence) suggests that the privacy-utility trade-off might deteriorate over much longer federation horizons in the absence of adaptive DP clipping strategies or privacy budget reallocation mechanisms. Future work should evaluate the robustness of the framework under more extreme data heterogeneity settings (e.g., Dirichlet α<0.5) and performance under stricter privacy constraints (ϵ<1.0), as such conditions are more representative of real-world clinical federated deployments.

## 7 Discussion

### 7.1 Comparison with baseline models

**Figure 15:**
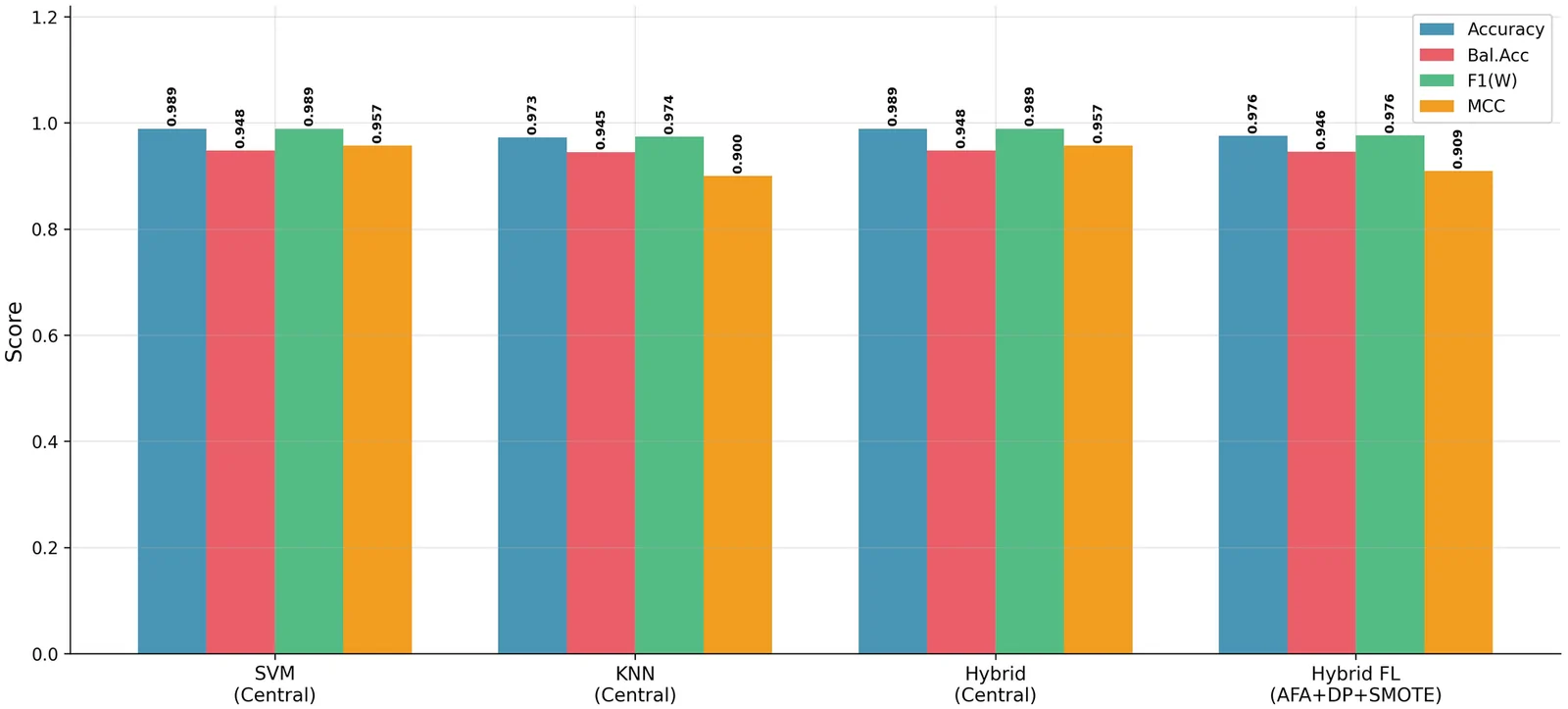
Ablation study model comparison.

The PPFH-SK architecture obtains a classification accuracy of 0.9755, a weighted F1-score of 0.9764, an MCC of 0.9089 and a Cohen’s Kappa of 0.9077, which is better than all centralised and federated baseline setups Figure 15. SVM employs margin maximization in high-dimensional MFCC spaces, a technique complementary to the instance-based local discrimination of KNN. The hybrid SVM-KNN ensemble integrates the strengths of the individual classifiers, compensating their deficiencies and achieving better decision fusion under non-IID conditions. The MCC and Kappa values are particularly important because to the class imbalance in the ICBHI dataset, as they offer measures of diagnostic performance that are immune to skew, something that raw accuracy does not. The macro F1-score of 0.9256 on the 368 test examples further demonstrates the discriminative power of local SMOTE augmentation across all six diagnostic categories.

### 7.2 Federated convergence and privacy-utility trade-off

PPFH-SK converges quickly and achieves near-peak accuracy at round 6 (Acc = 0.9755), and no client drift for all 20 rounds, which confirms that the AFA-guided aggregation can effectively suppress the model divergence caused by the non-IID Dirichlet partition in the usual FedAvg system. The temporary drops in accuracy at rounds 7 and 9 correlate with higher consensus entropies (0.774 and 0.664 respectively). However, the AFA mechanism helps immediately bounce back, which is a self-correcting stabilisation property of the process when dealing with diverse client contributions.

### 7.3 Advantages and limitations

The proposed PPFH-SK framework offers several practical advantages for real-world clinical deployment. First, it provides formal privacy guarantees through the (ϵ,δ)-differential privacy mechanism, while keeping evaluation metrics noise-free, so performance reporting reflects the model’s true diagnostic capability rather than privacy-degraded outputs. Second, the framework handles class imbalance effectively through client-local SMOTE, applied strictly within each client’s training partition to avoid data leakage. Third, the Adaptive Federated Aggregation (AFA) mechanism keeps training stable even under non-IID data distributions, evidenced by zero client drift across all 20 communication rounds.Finally, the model produces calibrated, interpretable per-class predictions, which matters for clinical trust and adoption.

A few limitations are worth being upfront about. The naive cumulative privacy budget (e=100 after 20 rounds) is higher than what strict clinical governance frameworks typically require, so tighter accounting methods (e.g., Rényi DP or zero-concentrated DP) would be needed before real deployment. We also acknowledge that this study does not include empirical model inversion attack experiments, so the practical privacy protection of the framework has not been directly stress-tested, only supported through formal DP guarantees. Similarly, no explicit heart-sound-removal stage was applied during preprocessing, which could be a source of residual noise in the acoustic features.The framework further assumes a trusted central aggregator, relies on hand-crafted acoustic features rather than learned representations, and was tested only in a simplified five-client simulation that does not capture real-world complications such as asynchronous participation, communication failures, or adversarial clients. Finally, prospective clinical validation on larger, multi-center datasets and real-world deployment studies remains necessary before the framework can be considered for routine clinical adoption.

## 8 Conclusion

This study has proposed a privacy-preserving federated learning framework for automated multi-class respiratory sound classification, which tackles three co-occurring clinical deployment barriers: prohibition on centralized patient-level audio aggregation under applicable data governance frameworks, extreme class imbalance endemic to real-world respiratory disease data, and statistical heterogeneity due to non-identical data distributions across distributed clinical institutions. Experimental results on the ICBHI 2017 benchmark dataset over 20 federated communication rounds tested in five-fold stratified cross-validation confirm the effectiveness of the suggested approach in all the assessment dimensions used. Future work includes adaptive per-round privacy budgets, cryptographic aggregation layer, deep acoustic feature learning to solve the residual misclassification, and projected multi-institutional validation.

## Data Availability

The original contributions presented in the study are included in the article/Supplementary Material, further inquiries can be directed to the corresponding author.
